# Making Sense of Making Meat: Key Moments in the First 20 Years of Tissue Engineering Muscle to Make Food

**DOI:** 10.3389/fsufs.2019.00045

**Published:** 2019-07-10

**Authors:** Neil Stephens, Alexandra E. Sexton, Clemens Driessen

**Affiliations:** 1 Department of Social and Political Sciences, Brunel University London, Uxbridge, United Kingdom; 2 Oxford Martin Programme on the Future of Food, University of Oxford, Oxford, United Kingdom; 3 School of Geography and the Environment, University of Oxford, Oxford, United Kingdom; 4 Cultural Geography Group, Department of Environmental Sciences, Wageningen University, Wageningen, Netherlands

**Keywords:** cultured meat, clean meat, cell-based meat, social science, sense-making, *in vitro* meat, naming

## Abstract

Cultured/clean/cell-based meat (CM) now has a near two decade history of laboratory research, commencing with the early NASA-funded work at Touro College and the bioarts practice of the Tissue Culture and Art project. Across this period the field, or as it is now more commonly termed, the “space,” has developed significantly while promoting different visions for what CM is and can do, and the best mechanisms for delivery. Here we both analyse and critically engage with this near-twenty year period as a productive provocation to those engaged with CM, or considering becoming so. This paper is not a history of the field, and does not offer a comprehensive timeline. Instead it identifies significant activities, transitions, and moments in which key meanings and practices have taken form or exerted influence. We do this through analyzing two related themes: the CM “institutional context” and the CM “interpretative package.” The former, the institutional context, refers to events and infrastructures that have come into being to support and shape the CM field, including university activities, conferences, third sector groups, various potential funding mechanisms, and the establishment of a start-up sector. The latter, the interpretative package, refers to the constellation of factors that shape or assert how CM should be understood, including the various names used to describe it, accounts of what it will achieve, and most recently, the emergent regulatory discussions that frame its legal standing. Across the paper we argue it is productive to think of the CM community in terms of a first and second wave. The first wave was more university-based and broadly covers the period from the millennium until around the 2013 cultured burger event. The second wave saw the increasing prevalence of a start-up culture and the circuits of venture capital interest that support it. Through this analysis we seek to provoke further reflection upon how the CM community has come to be as it is, and how this could develop in the future.

## Introduction

Cultured/clean/cell-based meat (CM) now has a near two-decade history of laboratory research, commencing with the early NASA-funded work at Touro College and the bio-arts practice of the Tissue Culture and Art project. Across this period the field, or as it is now more commonly termed, the “space,” has developed significantly while promoting different visions of what CM is and can do, and the best mechanisms for delivery. Here we both analyse and critically engage with this near-twenty-year period as a productive provocation to those engaged with CM, or considering becoming so. We write with the conviction that adopting a critical, self-reflexive approach to the history of one’s own field is an important activity for reassessing how the space came to be as it is, and recognize some of the possibilities of how it may have been otherwise. Being aware of how previous transitions within the field were shaped by socio-cultural framings supports a more rigorous interrogation of where the community may go next.

To be clear, this paper is not a history of the field, and nor does it offer a comprehensive timeline. It is also not a technical review of scientific progress over this period^1^. Instead it identifies significant activities, transitions, and moments in which key meanings and practices have taken form or exerted influence. We focus particularly on events where CM makers have met, presented and exchanged their ideas, and how these ideas have been contested in other spaces beyond the CM community. As such, the paper focuses upon cultural and political aspects of CM’s development as it has been made sense of as meat (or not) by different stakeholders. The paper was written in late 2018, revised in early 2019, and aims to identify key cultural-economic changes over the preceding 20 years. As critically-engaged social scientists who have been actively studying CM for over a decade, we see it as our role to render explicit political and cultural factors that have shaped and framed the development of CM, just as CM reframes and reshapes the cultural politics it inhabits. We do this through analyzing two related themes: the CM “institutional context” and the CM “interpretative package.” The former, the institutional context, refers to events and infrastructures that have come into being to support and shape the CM field, including university activities, conferences, third sector groups, various potential funding mechanisms, and the establishment of a start-up sector. The latter, the interpretative package, refers to the constellation of factors that shape or assert how CM should be understood, including the various names used to describe it, accounts of what it will achieve, and most recently, the emergent regulatory discussions that frame its legal standing^2^. In practice, these themes of institutional settings and interpretative work are intrinsically linked, and we recognize this in our writing while separating each into distinct sections of the paper.

Across the paper we argue it is productive to think of the CM community in terms of a first and second wave. The first wave was more university-based and broadly covers the period from the millennium until around the 2013 cultured burger event. The second wave saw the increasing prevalence of a startup culture and the circuits of venture capital (VC) interest that support it. While both waves are broad, there are key differences in the institutional context and interpretative package produced by each. This given, it remains important to stress this was a slow transition as opposed to a step change, with overlaps and continuities that remain, and an indebtedness of wave two to wave one.

In terms of structure, we first describe the social science research methods that inform this work, before dedicating a section each to the institutional context and then the interpretative package of CM. We then close with a final discussion that critically engages with the practice and potential of the institutional context and interpretative package as they stand today, and could be in the future.

## Methods

This paper draws upon three separately conducted research portfolios on CM. Stephens has been tracking the CM community since 2008, and has attended most major events in that time, as well as conducting 42 interviews with professionals working in the field between 2010 and 2013, and is currently conducting a second comparative set of interviews in 2018/9. He has also conducted media analysis of public reporting during this period. Sexton has researched the CM community since 2013, focussing particularly on activities based in and around the San Francisco Bay Area in California, US (otherwise known as “Silicon Valley”). She is engaged in ongoing fieldwork within this region and has conducted 30 interviews with professionals directly working in and affiliated with the field between 2014 and 2018. She has also conducted qualitative analysis on the online narratives of the CM field. Driessen has studied the CM community as it emerged in public media and in a series of academic and public events especially in the Netherlands, since 2008. Together with Cor van der Weele, he has organized three workshop-discussion sessions at bioethics, social science and CM conferences, and also conducted a study with five focus groups of prospective consumers on CM (although this work is not reported here, see Van der Weele and Driessen, 2013, 2019).

Collectively the authors have engaged in frequent exchange of ideas across the last 9 years for Stephens and Driessen, and the last 5 years for all three. Specifically in delivering this paper, the authors conducted a sustained dialogue on the content of the theoretical and empirical account. However, due to the research integrity practices of each individual project, the authors did not share data (e.g., interview transcripts) so as to maintain their ethical commitments to their interviewees and research participants. Instead authors discussed their data anonymously and developed upon existing publications in building their analysis.

The paper draws upon multiple independently conducted studies. Two of these studies involved recorded interviews with human subjects. Both secured written informed consent from their participants through protocols that were reviewed and approved by their respective ethics committees. For Stephens’ work this was Cardiff University School of Social Sciences Research Ethics Committee and Brunel University London College of Business, Arts and Social Sciences Research Ethics Committee. For Sexton this was King’s College London Ethics Committee. Driessen’s philosophical contribution did not involve data collected from human subjects but was conducted in accordance with the procedures of Wageningen School of Social Sciences.

## The Institutional Context of CM

All practices require some range of institutions to support their cultural, economic, and spatial accomplishment. In this section we first analyse how the early CM community emerged largely within a biomedical academic context and worked to develop institutional mechanisms to better support their endeavors. We then document a shift toward the second wave signaled by an increased reliance upon a venture capital fuelled start-up culture.

### Life in the University: The First Wave CM Community

Before the first wave of collective efforts to produce CM there were a small number of pioneers conducting laboratory work around the turn of the millennium. Two projects in particular—one in bio-arts and one funded by NASA—were both independently producing tissue around 2000-2001. One of these was conducted by Benjaminson et al. (2002). Morris Benjaminson first became aware of the concept in the mid-90s from his cousin, a chief food inspector at the New York City Department of Health. Having an existing track record of securing funding from NASA, he later submitted an application to their Small Business Innovative Research programme, which NASA funded. During the project the team cultivated goldfish explants to increase their size. It was inspected, smelt and described as “acceptable as food” (Benjaminson et al., 2002 p. 885). Importantly, the focus of this work was entirely to develop a muscle protein production system to support meat production during long-term space travel. They subsequently also grew chicken muscle (Wolfson, 2002), but NASA chose not to fund a subsequent research application as long-term space flight was a low priority.

The other early project to develop, and this time eat, CM was by bio-artists (Catts and Zurr, 2013) through their Tissue Culture and Art Project (Catts and Zurr, 2002). Initially developed during a residency at Harvard Medical School in 2000, the artistic project produced semi-living steaks from pre-natal sheep cells, before later growing steaks from living frog cells. The steaks were eaten at the world’s first CM dinner party as part of their Disembodied Cuisine exhibition at L’art Biotech, Le Lieu Unique, Nantes, France, in 2003. This provocative work sought to engender discussion about the transgressive status of the tissue, as the frog muscle was consumed with the live frogs from which the cells were sourced also sitting at the dinner table^3^.

The early 2000s saw a number of scientists who formed the first wave of research on CM (although it was mostly known as *in-vitro* meat at the time), with key figures including Vladimir Mironov, then of the Medical University of South Carolina (MUSC), Douglas McFarland of South Dakota State in the US, and Henk Haagsman of Utrecht University in Europe. Mironov and McFarland co-authored the first academic CM review article with Edelman et al. (2005). Edelman had previously written a review article in 2003 during a science journalism course in Wageningen, the Netherlands. Matheny had been inspired by the NASA work and was in the process of establishing a non-profit supporting the sector, which came to be the now well-known New Harvest, which at that time gave 100% of donations to research at universities.

Haagsman was Professor of Meat Science when in 2001 he was approached by another early pioneer of the community, Willem van Eelen, who had already secured a 1995 patent for *in-vitro* meat production. Early coverage of CM often revelled in Van Eelen’s colorful life story, citing his time in a World War II Japanese internment camp as key to the genesis of his thinking (Spectre, 2011). Van Eelen was an entrepreneur and a larger-than-life character who convinced initially skeptical Haagsman to seek funding for research. After 4 years of trying they secured around €2m for a four-year project based at Utrecht, Amsterdam, and Eindhoven Universities. The money came from a small interdepartmental programme of the Dutch government called the Programme of Sustainable Food Systems (PSFS). This is pertinent, as they are not the typical funders of Dutch research on tissue engineering or food science, as those groups had remained hesitant to fund CM studies. The project explored embryonic cell line development, algae-based culture media, and cell stimulation methods (Boonen et al., 2010; du Puy et al., 2011). Among the team members were Mark Post, and meat industry contact Peter Verstrate, who would go on to develop the cultured burger after this first project completed; meanwhile Haagsman led a second CM PSFS grant.

During this period other university laboratories pursued projects, notably Tor Lea and Stig Omholt at the Norwegian University of Life Sciences who grew fat and cartilage from pig umbilical cords, and Julie Gold’s group at Chalmers University in Gothenburg who worked on bioreactor designs and adhering muscle cells to starch cells. Bioethicist Welin (2009) was also central to the Swedish group, articulating the moral value of CM and pursuing research funding. He was one of a broad range of social scientists, artists and designers active at this time who offered support, raised questions, or suggested speculative futures through a broad set of analytical frameworks (King, 2006; Hopkins and Dacey, 2008; McHugh, 2010; Stephens, 2010; Van der Weele, 2010; Driessen and Korthals, 2012)^4^.

Further strengthening the role of the third sector in the community, in 2008 People for the Ethical Treatment of Animals (PETA) offered $1m for the first group to sell *in-vitro* chicken that was indistinguishable from livestock chicken in 10 US states. The campaign was fronted by PETA President Ingrid Newkirk, although a key driver of the campaign within PETA was Bruce Friedrich, who later co-established CM and plantbased meats advocacy group the Good Food Institute (GFI). Scientists within the CM community at the time felt the prize was a little gimmicky, as no one was close to producing tissue at that quantity or quality. Their preference would be to invest the money in a grant, which PETA subsequently did, funding Nicolas Genovese (who would later co-found Memphis Meats) to work with Mironov at MUSC on their “Charlem” (Charleston-engineered-meat) approach. Other third-sector groups were also active, including van Eelen’s own *in-vitro* Meat Foundation, and Austria-based Future Foods. By 2013, New Harvest had attracted sufficient funds to take on its first employee, its Executive Director Isha Datar, who steered the organization as the CM communities of the first wave transitioned into the second.

In general, the first wave CM community was typified by university-based projects (sometimes with industrial contacts) driven by environmental and animal ethics concerns but overwhelmingly hitting a wall when trying to attract funding from the traditional institutions that support university work. They generally saw their work as basic science, focused on building a knowledge base for subsequent innovators to develop products. The majority of the researchers involved came from biomedical backgrounds, often based in biomedical departments, and were fully aware of how some colleagues attributed an oddness to their pursuit of tissue-engineered meat. For many, it was a side-project financed through whatever funds could be found within existing budgets or through PhD and Master’s student projects, conducted as a secondary activity to their biomedical research and teaching. While they often attracted (sometimes frustratingly high levels of) press attention, they typically did not see their role as laying pathways for marketing or PR purposes, believing the technology to be too early-stage for PR to be a core emphasis of their day-to-day work. These roles, they believed, would be fulfilled by a different type of professional who would enter the field when the technology was better understood. This meant that across the community there were more diverse opinions about exactly what CM is or what it could be, with more openness than today both to the possibilities for whether it would be meat or a meat alternative, and the realistic opportunity for its world-transforming potential. Privately expressed, the dominant attitude tended more toward a sense that “we think this could have an incredibly positive impact so it is worth pursuing, but we would need to wait and see,” compared to the more confidently-asserted notion that “this will definitely deliver benefits in multiple ways” that we see in 2018. For some in the first wave, an uncomfortable tension remained between the visions they were asked to provide for what they saw to be media hype, and the realities they knew of their limited budgets and early-stage technology.

The biggest challenge facing the first wave of CM was funding. Many research proposals were submitted to governmental funding bodies in multiple countries, but met with no success. One core barrier was that CM remained a fundamentally novel concept that blurred existing categories. It did not fit well with funders that usually support tissue engineering research, because it was aiming to produce food. Equally, it did not fit well with funders that usually support food research, because it was tissue engineering. The ambiguity over its status meant it sat uncomfortably along the established disciplinary lines of university funding mechanisms. While we cannot evidence this, we hypothesize another barrier to attaining government finance was that the funding bodies may simply not have believed CM was a viable technology, more akin to science fiction than science fact, and possibly even that CM was stigmatized as an oddball science. During the first wave, CM’s unusualness remained central to how it was understood.

Then, famously in 2011, Mark Post of Maastricht University was interviewed in *The New Yorker* saying the technology was already available to grow muscle cells into animal protein in the lab, if the money was available to do it (Spectre, 2011). Within weeks an anonymous donor had offered the money, and Post was developing a programme to deliver the world’s first laboratory-grown burger. By 2013 the financial donor was revealed as Google co-founder Sergey Brin during the press conference in which the burger was cooked and tasted. It provided what remains the defining moment for the emergence of CM technology, setting in place a coherent vision for what CM is and what it can accomplish that has remained the robust and dominant account within the CM collective imagination. By staging the public eating of CM, the burger event asserted its realness, and realness as food (O’Riordan et al., 2017; Sexton, 2018). It set the template for making sense of making meat, as meat as we know it, and a technology designed for environmental, human health, and animal welfare benefits. The PR sensibility of the event brought a new aesthetic to the field, focused on style, slickness, and confidence. It was also significant in signaling a shift toward funding from commercial but mission-based Silicon Valley and Bay-area sources. In this regard, it sowed the seeds for the vision and economic underpinning of CM’s second wave.

### Conferences and Events

Key to the emergence of a CM community has been a set of events that have functioned to both bring people together and symbolically mark new stages in the field’s development. International events of this type started with an April 2008 meeting called the “First International *In-vitro* Meat Symposium,” at the Norway Food Research Institute, Ås. The three-day event hosted over fifty people discussing large-scale production, mission-critical challenges, R&D and feasibility. While these themes remain central today, few of the 2008 attendees would be known to a new entrant of the CM space in 2018. Jason Matheny, then and now of New Harvest, spoke at the event, as did Willem van Eelen, describing it as “a dream coming true.” Largely organized by the Dutch *In-vitro* Meat Consortium and colleagues in Norway, the symposium sought to enact community and, by taking the form of an academic event, usher in a new disciplinary trajectory.

It would be 3 years before the next international event, a European Science Foundation (ESF) funded exploratory workshop titled “*In-vitro* meat: Possibilities and realities for an alternative future meat source” held in Gothenburg, Sweden in late summer 2011. Stephens was one of around 30 attendees, many of whom had never previously met those from other countries. This event was notable as the first presentation to the emerging community by Hanna Tuomisto of her now well-cited life-cycle assessment of CM, a study funded by New Harvest (Tuomisto and de Mattos, 2011). The symposium is also remembered as the time the field decided to adopt “cultured meat” over “*in-vitro* meat.” Such accounts overstate the agreement around the room at the time, as discussed later in the section on nomenclature. It was in the months leading up to this event that Mark Post announced plans to produce the burger, and Mironov later announced the first tasting of his Charlem would be at the Gothenburg event. However, neither were able to attend the workshop. Post was absent due to ESF rules on funding limits to prevent over-representation of any single country at their events, as numerous others from the Dutch consortium were already confirmed, while Mironov had been suspended by MUSC for unacceptable behavior unrelated to CM (Saenz, 2011), although Genovese attended in his place.

The following year, 2012, the community further sought to establish the academic representation of the field at two events in which CM-related panels were hosted at major scientific conferences. The first, in February, was a panel organized by Genovese and funded by New Harvest titled “The Next Agricultural Revolution: Emerging Production Methods for Meat Alternatives” at the Vancouver, Canada, meeting of the American Association for the Advancement of Science (AAAS), and included Pat Brown (Impossible Foods), Mark Post, and Genovese himself. Later that year, in September, a panel on “Tissue Engineered Nutrition” was hosted at the Tissue Engineering and Regenerative Medicine International Society (TERMIS) conference in Vienna, Austria, an enormous conference on biomedical research. Speakers included bio-artist Ionat Zurr on the first consumption of CM in 2003, Mark Post on the plans for the burger, and Modern Meadow co-founder Gabor Forgacs, then the first start-up focused on CM. Notably, at the presidential plenary, the panel was name-checked as a distinct and unusual addition to the portfolio of work conducted under the rubric of tissue engineering. These panels were devised to give visibility and legitimacy to the still-then unusual technology of CM within the established academic realm by situating it as equal with the diverse range of other panels. Perhaps most noteworthy here is that as well as being the first panels of their type, to our knowledge, at the time of writing these were also the last. While we know of academic social science conferences with panels on CM, the articulation of technical knowledge on CM has moved away from sessions at large academic conferences to other forums.

A major moment came in October 2015 when Maastricht University, New Harvest, and Limburg-based technology support platform Brightlands hosted the “First International Symposium on Cultured Meat.” The event adopted the form of an academic conference, although the speakers came from a wider set of backgrounds. With around a 100 attendees, it combined speakers working on CM from laboratory and social science perspectives, along with others from biomedicine working on biomedical tissue engineering challenges with relevance to CM. It drew heavily on the legitimacy of the cultured burger and the Maastricht group’s role in it, with large photos of the burger prominently displayed in promotional materials. The significance of the event was not lost on those who attended, and was reiterated multiple times by the organizers. This first international event was a milestone in the emergence of the field. Although it wasn’t the first international event. It was either the second or the third, depending upon what was counted. But perhaps what was key here was that it was a significant milestone for the “field,” not the “space.” The trajectory aspired to by the organizers was still an academic, disciplinary mode of organization. The event featured a discussion on the establishment of an international CM society, modeled upon other academic societies, although the perspectives on the utility of this varied across the room, and we are not aware that the idea was pursued further (see also Jönsson et al., 2019). The conference was also a key networking moment as many met others in the field for the first time. It was also a key moment of inspiration for others, and we know of two CM start-ups today founded by individuals who attended Maastricht out of curiosity, but left with the seeds of a future career change.

2016 saw the New Harvest conference, branded as the “first-ever cellular agriculture conference,” in San Francisco in July. Unlike those prior meetings, this event did not adopt the academic conference as its model. Instead of lone-presenters reporting research findings, it featured panels and interviews on a range of topics. Conspicuously stylish in its aesthetic, this was an event to support the CM “space,” as opposed to the CM “field.” It captured the shift in the center of gravity of the community away from the university-based disciplinary activity of the first wave to the start-up culture and its distinct ways of acting, being, and looking of the second wave. That’s not to say that New Harvest was turning its back on universities at this time. Indeed, the event saw announcements of New Harvest funding in their university-based Research Fellows scheme. However, the culture was shifting, and this event symbolized and instantiated that shift.

The Maastricht and New Harvest events became annual, and other countries witnessed broadly similar events, such as the May 2017 Modern Agriculture Foundation event in Israel, in March 2018 the “1st Cellular Agriculture Conference in Japan,” and in 2016, 2017, and 2018 the Cultivate events in the UK. In the US, the Cultured Meat Symposium and GFI launched their own respective conferences in 2018, both in the San Francisco Bay area.

The establishment of conferences and meeting spaces has been both practically and symbolically important for the community. Events such as these make the notion of a community meaningful, as group photographs of attendees are circulated afterwards as evidence of their communing. These early events attained a sense of fanfare that ushered in this new community and the new technology they support. This is evident in the repeat assertion of the “firstness” of the events, be it the first for CM, for Cellular Agriculture, or in a new territory. In the early days the work of hosting an actual physical event lent credibility to the view that CM was an actual physical technology, and fought against the science-fiction label in an attempt to show it as a serious scientific and academic enterprise. Yet in the chronology of these events we also witness a shift from largely academic points of reference to those of a broader “space” typified by diverse cultures from entrepreneurship, the third sector, as well as those in universities.

### The Second Wave CM Community: Rise of the Start-Ups

With public funding generally unavailable at such a nascent stage of the technology, many CM developers sought out (or were approached by) wealthy philanthropic individuals and the investment streams associated with high-tech ventures. This shift became a key feature of what we here identify as the second wave of CM research. Technology accelerator programmes are one such model that has germinated a number of leading ventures in CM, including the first start-up active in the space, Modern Meadow, founded by Gabor and Andras Forgacs. While they already had start-up experience with the successful launch of biomedical 3D-bioprinting company Organovo, they opted to go through the 2013 programme of Singularity University, a tech business incubator in Silicon Valley aimed at breakthrough technologies to address humanity’s global challenges. At the time the Forgacs focused on both meat and leather, having eaten fried CM during a 2011 TEDMED talk^5^, and then producing CM “steak chips” in 2014, before focusing primarily on leather.

The typical structure of a tech accelerator involves cohorts of entrepreneurial teams who apply with an idea that they wish to develop into a for-profit business. The teams spend an intensive period of usually 3–4 months in residence at the accelerator, during which time they are given physical space, technical resources, seed funding and networking opportunities in exchange for an equity stake in the company. The programmes usually culminate in a “demo day” where the teams showcase their prototypes and pitch their business potential to an invite-only audience of investors, business personnel and media. The model was developed within and for the information technology industry, but has been extended to other sectors, in the case of CM most notably by IndieBio. Based in San Francisco and operating under the umbrella of investment management firm SOSV Ventures, IndieBio claims to be the first accelerator in the world specifically focussed on facilitating the upscaling of biotechnology companies. IndieBio’s first cohort included Clara Foods, a company formed through New Harvest that uses cellular agriculture to produce animal-free eggs whites, which quickly raised its seed fund of $1.7m. Following this success, IndieBio sought out potential CM start-ups to join the programme, resulting in Uma Valeti and Nicholas Genovese’s Memphis Meats entering their second intake. By demo day they were showcasing footage of the world’s first CM meatball, and went on to raise $17m in their Series A funding round^6^. Other CM IndieBio graduates are Finless Foods and New Age Meats, among other cellular agriculture companies.

The concentration of CM start-ups within Silicon Valley has in large part been a result of the region’s growing institutional interest and investment in food technology over the last decade, of which IndieBio is just one part. “Food tech” has become a distinct genre of entrepreneurial activity within the region’s high-tech ecosystem. It has featured as a frequent topic of industrial conferences and been heralded as a new frontier of Silicon Valley disruption; the hopeful successor of the clean energy movement that, for many in the Valley, failed to deliver on its (shortterm) promises and profits. During Sexton’s fieldwork in the region it became clear that a shift was underway concerning which global problems and solutions mattered to the Valley community. A common format for budding start-ups when pitching their ventures is to identify the explicit problem(s) that their innovation will purportedly solve. What is considered a problem and solution in the region are subject to particular conditions and rapid shifts in the attention economy of the Valley milieu. Sexton was told that previously “global warming” had been a big “world problem” that would appeal to mission-driven investors at the time, yet “global food security” has since become the new issue for start-ups to tackle^7^. In part a response to and driver of this broader shift in institutional interest, some of the region’s established VC firms have over the last decade expanded their investment interests to food technology ventures, including those developing CM. In addition, a more recent evolution has seen a number of new VC firms founded with a specific remit of funding early-stage food-tech ventures developing animalfood substitutes: examples include New Crop Capital, Stray Dog Capital and Kale Invest. The locations of these firms range from Silicon Valley to other parts of the US, as well as in Europe and Asia.

Other recent CM ventures have their origin stories closer to academia. Mark Post’s Mosa Meat (Netherlands), Aleph Farms (Israel) and Cellular Agriculture Ltd (UK) are examples of companies routed in, or with strong links to, academic biomedical research programmes, often with lead researchers acting as CSO, or Scientific Director in conjunction with their existing university roles. There are also a number of universitybased projects that do not have links to specific companies. New Harvest have long funded university-based work, and in 2018 GFI also announced funding for a set of projects. Subsequently, the shift toward wave two has not seen the end of university-based programmes, as by our reckoning there is more university laboratory work in 2019 than there was in 2009, but the universities have lower media profiles and incomes than the startups, and operate increasingly in relation to the language and aesthetic of the start-up space.

Other funding mechanisms have also been pursued. Israelbased company, SuperMeat, launched a crowdfunding campaign on IndieGoGo early in its formation, and has since gained seed funding from a number of VC firms within the US, some of which are solely focussed on animal-food substitutes. It was also reported in 2017 that the Chinese government had signed a $300 million trade agreement with SuperMeat, Aleph Farms (then Meat the Future), and another Israeli CM company called Future Meat Technologies. The deal was reportedly motivated by China’s current strategy to reduce carbon emissions and secure a safe future protein supply. Government-funded agencies in Singapore and Japan have also participated in recent funding rounds of cellular agriculture ventures (Friedrich, 2018b; Lee and Koh, 2019). By 2018, Mosa Meat revealed a significant funding round had been secured from the investment arm of German pharmaceutical company Merck, and leading European meat processor, Bell Food Group. This period has also seen an increase in Intellectual Property activity, as companies including Memphis Meats and Supermeat have filed patents on CM-related technology.

Investments from multinational (food) corporations are indicative of a pivotal shift in the institutional support of the CM sector. Until 2017, the bulk of investment secured by CM startups had come from wealthy philanthropic individuals, early-stage VC firms, and Valley accelerator programmes. Since 2017, the investment rounds of CM companies, and indeed the wider movement of recent alternative protein ventures, have made global headlines for including some of the biggest names in the conventional livestock industry, such as Tyson Foods, Cargill, and PHW. These developments represent strategic investments by the major incumbent players to keep track of the emerging sector; to ensure they are the disruptors, not the disrupted. Alternative proteins including CM are also beginning to attract attention from the global investment community beyond venture capital firms. The FAIRR Initiative, a ‘collaborative investor network’ established by the UK-based Jeremy Coller Foundation, has been a key player in consciously engaging the investment community—particularly those operating multi-billion dollar pension and asset management funds—on the environmental, social and governance risks posed by intensive livestock farming. One of the recommendations promoted by FAIRR has been for active investment in alternative proteins, including CM (FAIRR, 2018). The increased involvement of established food, pharmaceutical and investment companies can be associated with a softening of the rhetoric around disruption by some in the space, with GFI shifting to using “transforming” animal agriculture over “disrupting” it (Centre for Effective Altruism, 2017), and Memphis Meats suggesting the Silicon Valley approach of “move fast and break things” is an “awful” way to produce food (Watson, 2018a).

However, one company that still looks very attached to the San Francisco disruptive food tech space model is “JUST,” formally Hampton Creek, who in summer 2017 announced they would release the first commercially available CM product before the end of 2018. Echoing the 2003 frog-meat bio-arts project of Catts and Zurr, JUST released a video showing their team eating chicken nuggets in a sunny garden as a live chicken, the purported source of the cells, walks around them. The 2017 announcement that JUST planned to enter the market by the end of 2018 seemed incredibly bold, even by the standards of optimism and ambition of the second wave start-up sector. As we finalize this paper in early 2019, it is clear JUST missed their target. The reason, they suggest, was that regulatory approval could not be secured in time in any of the number of countries they approached. Currently they anticipate launching by summer 2019 (Watson, 2019). Should this happen then the related regulatory approval and media coverage could form another key element in framing how CM can be understood and how it can be contested. The short timeline to market proposed by JUST was an acceleration of previous estimates, and the impact of such increased expectations concerned some in the community at the time. Anxieties over an early launch remain as we write. Media reports tell how Eric Schulze of Memphis Meats addressed other start-ups during the 2019 “Industrializing Cell Based Meats” conference, arguing that the first to market bears a responsibility for the perception of the rest. He is quoted as saying “we’ve seen from other areas in the food industry how subpar early products can stigmatize an entire category for decades… or botched regulatory strategies can result in years or even decadeslong delays,… what one company does or says will affect us all” (Watson, 2019). How JUST’s product will be presented, how much it will cost and its physical properties, are all questions waiting to be answered.

In early 2019 the CM space is characterized by significant optimism, with new companies coming out of stealth mode almost monthly. Memphis Meats raised a disclosed amount of $17m in the first Series A funding round of any CM venture, a significant feat for a company without a steady revenue stream. Compared to wave one, the CM start-up community has more money, looks more confident, stylish, and a little younger. It has also been characterized by the rise of Silicon Valley as an important regional hub of CM activity and hype. By 2018 some companies were able to pick and choose which venture capitalists they took seed funding from. This period also saw the beginnings of start-up companies located beyond the US in Asia (e.g., Integriculture and Shiok Meats) and Europe (e.g., Meatable and Higher Steaks). However, seed-level venture capital is, of course, highly speculative, and VCs expect a high proportion of their investments to fail. No doubt some CM companies will meet this fate, perhaps with some staff subsequently recruited by those that survive. The optimism of wave two could be tested as more seek Series A funding, and the patience of investors could be tested if projected milestones for delivering scaled-up operations are missed.

## The Interpretative Package of CM

The first-wave CM community knew well that most people thought their work was unusual, a genuinely strange concept more associated with science-fiction than edible reality. As a profoundly novel practice, the real-world production of CM had no existing framework for making sense of the tissue. Those in the community needed to engage in the cultural work of developing sense-making mechanisms that not only rendered it knowable, but also desirable. Ambiguity over CM’s status has been a persistent feature of the first 20 years of its development, evident in its media coverage, the claims to its naturalness and potential, early regulatory discussions, and perhaps most enduringly, within the so-far continuous discussions as to what it should be called. We explore each in turn.

### Nomenclature

Central to the interpretative package of any novel entity is what it is called, and this has certainly been true in the case of CM. Readers will have noticed our use of CM within this paper to strategically avoid siding with cultured, clean, or cell-based meat, the three main contenders at the time of writing. But even focusing upon these three examples hides a broader complexity and a politics that stretches across the 20-year history discussed here, and will likely stretch further into the future. It is important because words do work. They invoke meanings, potentials, futures, networks, and identities. Here we discuss naming in the Anglosphere, but we note that there are additional complexities and cultural-specificities as the same issue is addressed in other languages. The NASA funded work used the terms “*in-vitro* edible muscle protein,” while the provocative artworks of Catts and Zurr opted for “semi-living steaks.” However, the first communally-used term was “*in-vitro* meat,” used by the Dutch *in-vitro* Meat Consortium, van Eelen’s *In-vitro* Meat Foundation and a number of early academic publications (Langelaan et al., 2011). Literally “in glass,” the term *in-vitro* comes straight from an academic repertoire evoking the world of the research laboratory, reflecting the center of gravity within the field of wave one.

*In-vitro* meat was the dominant term from at least as early as 2005. A narrative has built up within the CM community that this changed in 2011, specifically during the Gothenburg “European Science Foundation Exploratory workshop in *In-vitro* Meat.” However, Stephens attended this meeting and we argue this narrative writes out a level of hesitancy among many present to adopt the proposed “cultured meat,” as the merits of this and “*in-vitro*” were discussed collectively. More than “a name” was at stake in this discussion, as the very status of the technology and the participants’ research programmes were implicated. Being regarded as the scientifically accurate phrase, the term “*in-vitro*” remained the preference for some participants. That said, it was recognized that the term could alienate some people in a way less likely with “cultured,” which benefitted from the combined meanings of “cell culture,” “fermented,” and “artistic or classy” (cf. Datar, 2015). “Cultured” was embraced by some who deemed it more appealing to potential consumers, and was accepted by others because the cell-culturing element meant it remained within a scientific repertoire. However, it was clear many in the room would not accept a term that distanced the science altogether; as noted above, many present identified themselves as science researchers, not marketers, and that any name would need to be of the scientific realm, with a subsequent renaming of the technology to be led by a different set of professionals (perhaps in public relations) in a future moment once the technology shifted from research to commerce. As such, through the exchange on naming, those partaking in the field were defining themselves, the proper category of work they engaged in, and the status of the technology and the tissue itself.

While the 2011 meeting was not a decisive turning point, it was certainly an important moment. The switch to “cultured” developed over the coming months and years, finally becoming stamped as the phrase of the moment with its use at Mark Post’s cultured beef burger event. However, it is worth noting that at the time of announcing the burger in 2011 Post was using the term lab-grown meat, and even just 2 months before the burger launch, in his TEDxHaarlem talk, Post still did not say cultured, although it did feature on one of his slides. Returning to the 2011 Gothenburg event, it is also worth noting that many of those most wedded to the term *in-vitro* meat did not switch to cultured meat, but became increasing less visible in the field, some leaving altogether.

Then, in late 2016, clean meat became pushed more strongly, most notably by GFI, who used the term frequently at the 2016 Maastricht Cultured Meat conference, and explained why in a September 6th blog post. Here, Friedrich (2016) articulates the preference for clean meat based upon the term’s accuracy, its synergy with clean energy, and its capacity to lead conversations onto the positive aspects of why the meat is clean. Clean meat then became the title of Shapiro’s (2018) influential book. By May 2018 Friedrich published another blog post, documenting the increasing prevalence of the term among some start-up companies, media publications, and google searches (Friedrich, 2018a). The post also reasserted the term’s appropriateness, while commenting on four surveys of potential consumers showing their preference for “clean” over “cultured.” However, the switch to clean was far from total, with notable exceptions being Mark Post and New Harvest. Indeed, there is an extent to which an individual’s preferred term sometimes reflected their greater alignment with GFI or New Harvest.

Then, during the summer 2018 discussions around FDA or USDA regulation (discussed in more detail later), Memphis Meats used the term cell-based meat. This phrasing, they believed, could bring a level of alignment between the needs of regulators, companies in the CM space, and the livestock industry who have largely criticized the term clean meat for its suggestion that meat produced by slaughtering livestock is unclean. Cell-based meat was also used in the joint letter signed by Memphis Meats and the North American Meat Institute (NAMI) suggesting a combined FDA/USDA regulatory approach. Following this, at a private meeting after the inaugural GFI conference in September that year, a group of company founders decided to establish a trade association for the space, adopting the term cell-based meat to describe their future product. The FDA and USDA themselves, however, have tended to use “cell-cultured,” typically followed by “product” or “food product,” and not using the word “meat” at all.

At the time of writing it is unclear whether cell-based meat will stick in the way *in-vitro*, cultured and clean have. It is clear there remains resistance as some continue to assert the strengths of clean meat, while others still retain cultured and others again avoid adopting any specific phrase in their promotional materials. However, there are a set of learning points here. First, we would argue that the ongoing renaming and discussion over terminology is likely to continue at least for the short-term. It reflects both a fundamental ambiguity about what the tissue is and the cultural-institutional space it may come to inhabit in society, as well as the micro-politics within the CM community itself. Second, we note these naming disputes do not just reflect ambiguities over what the tissue is, but renaming efforts also seek to *intervene* in this meaning-making process by framing what it can accomplish, and by asserting it is meat. Third, there is an underlying identity politics in choosing preferred names, that relates to assumptions about the correct mode of activity—and the appropriate audience to be addressed—for the community at a point in time. Within this, there can be a second level of identity politics around who supports whom within the community, and what term they prefer. Fourth, in late 2018 we are witnessing perhaps the greatest level of pluralization of terminology since *in-vitro* meat became the established term. There are now clear and sometimes entrenched camps behind the cultured, clean, and cell-based nomenclature, as well as others adopting none of these. Equally, we are also seeing single actors using multiple terms, and may see the emergence of potential trade names such as JUST Meat and Memphis Meats, that could seek to advertise through brand names instead. This reflects similar strategies by the plant-based Impossible and Beyond Burgers which both avoid generic descriptors such as veggie burger. We may increasingly see the differentiation of terminology as one term is preferred for institution-facing regulatory work and another for consumer-facing promotional work. Collectively, these observations point to the significance and complexity of naming in the interpretative repertoire of CM, as doing so works to define appropriate roles for the community and what their tissues can accomplish. And as our final comment on this, we should note one constant across this flux and plurality of CM terminology within the community: the use of the word “meat” has remained. We would argue this signifier does more work than whatever term precedes it, and it is telling that the community has rarely deviated from it, since the early NASA and art practice work. While many in the community argue it should be called meat because it is meat, we again stress the contingent nature and interpretative flexibility of meaning for any words, no less those used here. While meat has been robustly sustained within the community, it remains imaginable that this may be challenged if actors decide that provenance is more important than biological physiology in meaning-making, accuracy and labeling. Indeed, as we will show, such challenges have already been made.

Other elements of the emergent discourse can also be analyzed in a similar way, such as umbrella terms “cellular agriculture” or the “post-animal bioeconomy.” In the 2018 GFI conference it was suggested that bioreactors are better termed “cultivators” to align more closely with the terminology of the food sector. Detailed analysis of these extends beyond the limit of this paper^8^, but we note such terms are used to assert meanings, draw boundaries that include some (and exclude others), and to attract the support and approval of various groups of actors. Words matter, and that is why the continued negotiation of language has been an ongoing feature of the CM interpretative package.

### Normality and Transformation

CM researchers over the last 20 years have sought to develop a technology that initially strikes many as odd. They seek to present it as paradoxically both normal and transformative, describing it as both familiar yet facilitative of profound social change. This work has been deemed necessary to bring meaning to CM in a way that seeks to soften any discomfort people may experience with this novel form of tissue, particularly as a substance intended for eating, while also articulating the reasons for supporting it.

One key strand of the rhetorical strategy for asserting the normalness of CM has been ever-present since the early days of New Harvest: that the production method for CM reflects familiar (even artisanal) production methods of existing and well-trusted foods, most typically beer, bread, and cheese. New variants on this theme have arisen over time. During the later stages of wave one, Nicholas Genovese promoted the term “carnary” for a CM production facility, mirroring the brewery, or dairy as established food production operations (Notaro, 2011). More recently, New Age Meats presented the vision for a shared craft beer and CM production facility with similar machinery for meat and beer flanking each side of the room to show consumers the commonalities of the two. However, during the second decade of the emergence of a CM community we have seen another key strand of the rhetorical strategy articulated with increasing conviction: that CM is meat, real meat, exactly as we know it from traditional forms. Scientists in the first wave of CM research were more likely than those of today to admit ambiguity as to whether CM (or *in-vitro* meat as most called it at the time) really was meat, or a meat-like foodstuff. The term *in-vitro* meat was sufficient, for many at the time, in capturing both the similarity and difference between meat made through tissue engineering and meat from animal slaughter. The argument claiming CM should simply be called “meat,” because that is what it is, was less strongly pronounced then in comparison with the second wave.

The narrative that CM is meat, ‘just not in a cow’, we argue, became established as the central line for the community through the 2013 cultured beef burger event. In many regards, this was a key moment for articulating a coherent and socially-visible form of interpretative package for CM that has continued to frame much of the discourse five years later. Yet even here, in the pre-recorded film introducing the event, the issue of sameness and difference required addressing. During the piece, Post hypothesizes a future encounter in 2033 when a shopper enters the supermarket to find two identical pieces of meat. He then articulates differences between the two – one involved killing animals and has an eco-tax – before again stressing that they are exactly the same in taste, quality and price, although the CM product may be cheaper. This example captures a paradox that remains inherent within the CM interpretative package: those supporting it seek to assert it is both ontologically the same thing, while also being different in crucial ways^9^. This thinking is now well established within the CM community. CM needs to be recognized as meat to deliver upon its world-changing potential; if it is seen as a meat *alternative* then it becomes another case in a long list of meat alternatives that as yet have not convinced most meat eaters to give up slaughter-based food^10^. Asserting CM’s status as meat is implicated in its promissory narrative and has been a central component of it being made sense of as ‘food’ by its developers (Sexton, 2018).

The asymmetry here is that some components of the difference between traditional meat and CM are stressed, indeed celebrated, while others are denied or played down (Sexton et al., 2019). In particular, the components of difference that are stressed are promissory elements such as the environmental footprint and animal welfare impact, which are articulated as knowable and fact-like, a confidence that has often drawn legitimacy from the small number of life-cycle analyses that have been conducted to date (e.g. Tuomisto and de Mattos, 2011; Mattick et al., 2015). The components of difference that are played down are the radically different mechanisms by which CM is produced compared to livestock-rearing methods. These components of difference are, of course, inherently linked: the promissory elements are premised upon different production methods. Yet it remains culturally significant that sameness and difference are being constructed in a particularly nuanced manner intended to afford interpretations of CM being meat like any other meat. It is normal, yet, or precisely therefore, transformative (Sexton, 2018).

However, it is important to analyse the narrative that CM is meat as we know it. Little inspection is needed to see that this articulation operates by seeking to assert that the status and meaning of something is a function of its physical properties. Proponents of this frame describe the cellular and tissue compositional properties of CM, or potential future CM, to validate its sameness as traditional meat. However, physical properties are only one way of asserting meaning, and typically meaning-making involves the interweaving of multiple historically-situated ways of knowing. A clear alternative frame found widely in the food industry that would stress differences between CM and livestock-produced meat is provenance. The two clearly come from, and come into being, through very different mechanisms. Another way meaning can be ascribed to something is through what it does, or how it is used^11^. In this performative or practice based understanding, CM could be understood as meat if it is consumed as meat, and recognized as such by all parties involved, including producers, consumers, and governments. This of course gives some agency to the CM community in making it “meat,” but also makes clear the community alone cannot assert a decisive outcome. Writing on the distinct yet related category of “edibility,” House (2018 p. 83) warns against “seeing the construction of edibility as the responsibility of entrepreneurial strategy” alone, but rather as something that is “co-produced by a diverse range of actors” (see also Vialles, 1994; Roe, 2006; Evans and Miele, 2012; Sexton, 2018). Likewise, in establishing the “meat-ness” of CM, consumers and, as we discuss in the next section, regulators are just some of these wider actors that have a key role in CM’s meaning-making.

Also key to developing an interpretative package for CM has been providing articulations of what it will achieve. Such narratives have always been used to convince people of the case for supporting the technology (Chiles, 2013; Jönsson, 2016; Stephens and Ruivenkamp, 2016; Ferrari and Lösch, 2017). We would argue that in the first decade of the community’s practice they were also used to counter intuitions that CM was a frivolous, even ridiculous idea, by pointing to serious issues the technology could be positively associated with. Such narratives have a promissory character, in that they assert future benefits that are not achievable at the time of articulation, and request a level of faith that CM is technically feasible and able to drive social change in the absence of any material evidence. Subsequently, a diverse range of interrelated promissory narratives were developed that are now familiar to anyone with any experience of the community. This given, we can observe shifts in what form these promissory narratives take. The early work was exclusively focused upon space travel, before this largely faded from sight. Animal welfare and then environmental narratives became more visible, while more recently human health and food safety narratives have been stressed, particularly in relation to debates about antibiotics in the US (Sexton et al., 2019). And, of course, for those in industry, the prospect of gaining a potentially lucrative market share in the global meat industry has also motivated engagement (Mouat and Prince, 2018). As such, these promises are being used to drive support for the community. As part of this, recent years have seen the emergence of species-specific sub-promissory narratives that apply broader CM narratives to the particular farming contexts of their target species. Examples include Finless Foods’ focus on overexploited fisheries, as well as mercury and plastics in the ocean, and New Age Meats’ suggestion that industrially-farmed pigs are subject to four times the antibiotic use of cows per pound of meat. This emergence of sub-promissory narratives is in part a response to a need for start-ups to differentiate among themselves in order to attract venture capitalist support, as each jostles for funding and status. But viewing promises as driving funding alone would underrepresent the rich work future visions do in making CM knowable and desirable (Stephens, 2013). The story of CM is a story of framing links between the now and a realm of potential futures.

### Regulation

To date, the interpretive package of first and second wave CM has largely operated through the articulations of stakeholders and events *within* the CM space, with most framings being of a promotional nature. The sense-making of what CM is and what it can do has thus primarily been the work of an academic field turned nascent industrial sector attempting to stabilize and promote a novel endeavor. During 2017-18 the space matured enough from its “nascent” status to enter the broader consciousness of established (food) corporations and national regulatory bodies. This transition brought powerful actors from outside the CM space—both supporters and challengers—into the interpretative process of CM, leading to this novel entity being held up for scrutiny against existing regulatory frameworks and the political and institutional orderings of the incumbent livestock industry.

With such shifts we find new questions being asked of CM by a new collection of stakeholders. While the question of whether CM is “meat as we know it” has followed the sector throughout the first and into its second wave of development—albeit with varying levels of engagement-2018 saw this query take on new political and cultural meaning. Through the contestations of newly-involved stakeholders, mainly lobbyist and other interest groups of incumbent agricultural industries, the sense-making of CM has become entangled in competing value-laden ideals over what a protein food system should look like quite literally on the ground, and what (in)tangible services it should deliver (Sexton et al., 2019). Such disputes have sought to entrench the necessity of meat’s status on its physical connections to particular environments (i.e., outdoors), institutional landscapes (i.e., farms, ranches), and animal bodies that are reared and slaughtered in the “traditional” manner, as well as its cultural entanglements in the livelihoods and rural economies within these places. The most high-profile examples of these contestations have originated in the US. The state of Missouri made headlines for passing a bill in August 2018 prohibiting the use of the label “meat” for any products that do not come from a slaughtered animal reared through conventional methods (General Assembly of the State of Missouri, 2018). The bill has since triggered CM-advocacy group GFI, in partnership with the Animal Legal Defense Fund, the American Civil Liberties Union of Missouri and plantbased meat company Tofurkey, to file a lawsuit accusing the bill of protectionism and violation of multiple legal doctrines, including the First Amendment. In 2018 a similar bill was deliberated by the French government to restrict the use of the label “meat” and its related terminology (e.g., “sausage,” “steak”) solely to conventional animal-derived products (BBC, 2018). Proponents of the bill argue that such restrictions will guard against consumer confusion and protect the traditions and livelihoods of conventional livestock producers.

Parallel contestations have come from US meat lobby groups which have, over the course of 2018, filed several petitions and open letters calling on US regulatory bodies to explicitly exclude CM and plant-based products from the statutory definition of meat and related terminology (e.g., “beef”)^12^. A shared feature of their arguments has been the necessary connection between such products with reared animals and conventional producers [e.g., “actual livestock raised by farmers and ranchers” (NCBA, 2018 p. 1)]. However, the ontological ambiguity of CM has somewhat complicated these debates. The National Cattlemen’s Beef Association (NCBA) was among a few early contesters in 2018 who supported the inclusion of CM in the legal definition of meat products, but not “beef” (NCBA, 2018 p. 2). It was voiced within the CM space that this stance was politically motivated: the inclusion of CM under the statutory definition of meat would result in the sector falling under the regulatory remit of the USDA, a federal body that is seen as having close links with the incumbent US livestock industry. In response, many CM stakeholders supported the FDA acting as the sector’s regulatory body, on the grounds that the FDA would be more politically neutral in its assessments and that it possessed more relevant experience given its dual role in regulating food and biomedical products. A position of compromise was put forward by Memphis Meats CEO, Uma Valeti, and the president of NAMI. In a letter submitted to President Donald Trump in October 2018 they outline a framework that would see both FDA and USDA regulating CM, but at different stages of its product development (Watson, 2018b). A joint meeting of the USDA and FDA was convened later that month with a view to developing a combined system. In March 2019 the two agencies announced a formal agreement to share the regulatory oversight of CM products (USDA, 2019).

In the EU, a key market for the CM community, the use of GM techniques in some CM production methods remains a key issue of regulatory uncertainty. It has been suggested that the EU’s approval of GM-produced rennet (i.e., chymosin) could act as a precedent for CM products that include GM methods in their production. At the time of writing there is also ambiguity about whether CM will be classified as a novel food, a category which comes with its own more detailed, expensive and lengthy bureaucratic processes. As of early 2019, the message from UK-EU regulators in events we have attended indicate that CM will be classified as a novel food, and that its product labeling will be required to make explicit that it was produced in cell culture and is “different” from conventional meat (although the exact phrasing is not yet clear). Another area of uncertainty is how to label the “country of origin” for CM products, an issue which will likely depend on where the cells are sourced. The potential use of immortal cell lines could pose particular traceability issues within this context. Questions have also been raised in regulatory fora over the potential health risks CM could pose to consumers^13^. Even if CM ventures endeavored to comply with EU regulations as they currently stand, it is recognized by regulators that existing frameworks are not sufficient to deal with CM, and this may add significant time to the process as additions and refinements are made.

As with the other components of CM’s interpretative package, regulatory frameworks at multiple scales and across different geographies present further spheres through which the meaning of what CM is (and what it can do) is being shaped and contested. Such dynamics signal a key shift in this meaning-making process moving beyond the confines of the CM community: it is no longer a community engaged solely with itself but is now being required to engage with new questions and contestations from a range of stakeholders over what role CM should play (if any) in the future food system. As Sexton et al. (2019 p. 62) note, such disputes touch on longstanding hopes and fears over the meaning of “good food” and the “contested place of science, technology and capitalism in the ordering of postmodern societies.” So far, the contestations within the regulatory context have primarily concerned the labeling of CM, its safety, and the political, cultural and economic stakes associated with its legal inclusion in the category of “meat.” This question of whether CM can be legally classified as meat is only the first step along a range of different and, at least in some regions, long and expensive regulatory pathways for CM products. Indeed, as Jönsson et al. (2019) note, the question may be reframed from *whether* CM is meat to *where* CM is meat. As such, considerable uncertainty remains a prevailing feature of these deliberations, both regarding the status and implications of CM but also how existing regulatory frameworks should potentially respond.

## Discussion

Above we have offered an overview of the institutional context and interpretative themes that have characterized CM development during the first 20 years of laboratory work. We identified two distinct waves in which CM has emerged. The first wave was largely university based and embedded its practices within the format of an academic field. However, it struggled for money, despite repeatedly seeking funds from typical university funders for basic “*in vitro* meat” research. The technology, and the community of people who pursued it, retained an unusualness to many onlookers, with persistent ambiguities over what was being produced and why it was being developed. The second wave saw the rise of the start-ups, who, often backed with Silicon Valley money and Silicon Valley ideals, sought to recast CM’s unusualness as “transformational.” Embracing the disruptive paradigm (Morozov, 2013), these early companies and their supporters provided much slicker visions of how CM would change the world, and why the world needed changing. As such, the institutional and the interpretative are linked, as new funding sources opened up new research opportunities in companies, and new aesthetics for promoting the technology. In the process, increasingly the CM community has reframed the cultural politics it inhabits, reconfiguring what constitutes tissue engineering (to include food), and as part of a movement that reshapes what counts as meat, while also problematizing livestock production methods. Through the gradual move from wave one to wave two, and through the continuities and indebtedness between them, the CM community has increasingly asserted a shared vision for what its technology is and what it can do.

In the final part of this paper we aim to specify a number of themes and questions that in the coming years are expected to be crucial in defining the further development of (the space of) CM. We address several in turn to provoke speculation on the possible futures for CM and what may bring them into being, and to question often taken-for-granted assumptions in the CM discourse as it currently stands. As such, we look forward to the ways in which certain interpretations and infrastructures can be at the center of future debates about what CM could be and might achieve. We do so by exploring interpretations and infrastructures on three themes: future corporate structures; environment, landscapes and rural infrastructures; and global meanings and institutions.

### Future Corporate Structures

The space of CM is defined by the types of actors involved and the places and institutional settings where CM R&D occurs and is communicated. As we move from the academic enthusiasm of biomedical professors to a VC-funded and IP-driven start-up culture, questions emerge about which institutional structures will come to drive subsequent CM development. Potentially the companies and products that will emerge from the fierce competition of the incubator ecosystem, in which only a subset are expected to thrive, may not eventually challenge the concentration of power in the food system. Assuming CM technology develops successfully, there are a range of commercial trajectories for contemporary start-ups in what may become the CM “third wave.” These potentially include becoming new giants themselves; being incorporated into existing larger companies (either current corporate meat behemoths seeking to vertically integrate the industry, the even larger food multinationals, or emerging plant-based meat alternative companies); selling or licensing technologies to other companies; or remaining smaller, potentially niche, businesses. Considering how these and other possible routes for a potential third-wave CM community relates to food system power structures should continue to enliven both critical thinking in the area, and analysis of how promissory narratives, meanings, and practices are produced.

Another important consideration is what institutional structure may exist in a future third wave if CM technology is not developed successfully, or develops more slowly than some anticipate. Much depends on why planned progress would be halted or slowed, and how the diverse range of venture capitalists currently supporting the work respond. Interesting in this context would be whether any progress attained during the second wave is sufficient to entice governments and other supporters of public research to channel funds into a new wave of university-based work in support of future commerce (as is seen in many novel technological markets). Conversely, failure of the wave two start-ups might be seen to validate previous government funders’ decisions to stay away. Whichever of these diverse outcomes, we expect the emergence of a third wave would bring new institutions and interpretations, which will bring with them new issues for consideration.

### Environment, Landscapes, and Rural Infrastructures

The impacts of large-scale CM production on rural livelihoods and landscapes remains ambiguous. Current intensive livestock farming has been increasingly positioned (with ongoing contestations) as an environmental dead-end. Yet how and to what extent CM would transform the meanings and infrastructures of the countryside is unclear. It is often assumed the mere “disruptive force” of CM will solve the current ills of the global food system. But CM does not inherently address issues of social injustice, food sovereignty, productivist interpretations of global food security, loss of agrobiodiversity, and the concentration of power within the current food system. CM tends to be defined as clean, ethical, low-impact meat (Sexton et al., 2019), but making good on its environmental and ethical promises will depend on how efficient the process can become and what steps are involved in the chain of CM production, as well as the forms of infrastructure and economic means in place to enable it (materials, transport, energy source etc.). It is also dependent upon the motivations of those organizations that produce future CM (and their funders) as they may face tradeoffs between the most environmentally-friendly or socially-just production methods against the cheapest or safest.

Many CM actors have emphasized the devastating effects of feed production elsewhere—e.g., South American soy—for intensive farming, and the environmental destruction from animal production through emissions, land and water use, growing antibiotic resistance, and pollution associated with manure lagoons. The impacts of a dominant CM sector on global agriculture would however involve a reorganization of the role of animal production (including feed) and soil fertilization, which is easily overlooked when “intensive” and industrial “factory” farming is conflated with all farming methods involving animals. If CM can make good on the promise of reduced land use, it is still not certain this will result in scenarios that will produce more beneficial outcomes in environmental or indeed socially just ways. At the time of writing there are currently no regulatory mechanisms for supporting the transition of any land spared through CM development to projects of reforestation, rewilding, or other forms of ecosystem restoration and conservation, clean energy production or other uses intending to generate environmental benefits. It is also not clear that market mechanisms alone can be relied upon to deliver optimal environmental outcomes. Equally, these examples of land-use change are highly contested in terms of the types of social and environmental benefits they bring, particularly within different geographical and cultural contexts (Benton and Wellesley, 2018). The potential for CM to spare significant amounts of land from conventional agriculture (itself still a speculative promise), and the possible directions of land-use change this could trigger, remains a critical issue for further consideration by the CM community and external stakeholders. The frameworks of understanding that emerge with commercialized CM, and the institutions and infrastructures that are generated to support it, will be central to its impact on landscapes, livelihoods and environments.

### Global Meanings and Infrastructures

While there are a growing number of CM start-ups internationally, particularly in Asia, it remains an open question how CM would land on emerging markets where some aspiring middle classes are developing an appetite for meat (WEF, 2019b). Early studies on consumer attitudes beyond Europe and North America have indicated that acceptance of CM will depend on existing perceptions of what counts as meat, both in terms of the raw materials used (e.g., animal flesh, plant matter) and desired sensory characteristics such as mouth-feel and taste (Bekker et al., 2017). The cultural and political status of meat in different places, as well as established histories of meat abstinence (e.g., India), will also likely shape how alternatives are received into national and individual food practices (WEF, 2019a). Notions of “naturalness” and the acceptance of technoscientific interventions in food production have additionally been highlighted as important attitudinal drivers toward CM (Bryant and Barnett, 2018), both of which are subject to key regional and cultural differences. Moreover, we might speculate that the political landscape of different places will also play a key role in CM acceptance: for example, the prominence of environmental and welfare issues on political agendas and their explicit connections (or not) with conventional livestock production, and the makeup of regulatory frameworks will likely shape where CM activity expands over the coming years, and who its consumers, advocates, and contesters will be.

One concern about the emergence of global meanings and infrastructures should be whether CM comes to be part of a neo-colonial project that seeks to feed the Global South with the protein fixes of Northern corporate marketers (Sexton et al., 2019). Such a scenario risks ignoring the varieties of economic and cultural value afforded livestock production and consumption in the Global South, as well as the relative environmental footprints of its different systems (WEF, 2019b). It also risks the continued reimagining of Southern nations through the logics of the global free market. Such trends have been critiqued for advancing neoliberal interpretations of food security, which in many cases have been shown to exasperate the very issues of hunger, poverty and environmental degradation that motivated their introduction, as well as further entrenching political-economic imbalances across Northern and Southern geographies (Jarosz, 2011).

This given, livestock production in the Global South is highly differentiated in size, operation, and political-economic power. For example, middle-income nations in Latin America and Asia have some of the highest livestock numbers in the world (Robinson et al., 2014), with production ranging from small-scale subsistence farms or nomadic pastoralists to highly industrialized operations with animals numbering over 50,000^14^. As has been experienced in Northern contexts, concerns have been raised about the long-term ecological sustainability of industrialized livestock and animal feed production in emerging economies (FAIRR, 2016 p. 11). Specific concerns such as food safety—an issue of considerable public concern in China (Garnett and Wilkes, 2014)—may also continue to plague the reputation and long-term economic sustainability of intensified conventional production. Indeed, initiatives such as FAIRR are actively applying pressure through investment channels to redirect protein production away from factory farm facilities on account of their rising environmental, social, governance—and thus economic—uncertainties (FAIRR, 2016). It is possible, therefore, that CM could become an attractive alternative for large-scale meat producers in some Southern nations. Yet in the same way as Northern contexts, it remains to be seen whether this would assume a wholesale replacement of meat production or rather come to exist *in addition* to conventional livestock operations.

## Conclusion

We have detailed how, during nearly 20 years of laboratory work, CM has shifted from a university-based first wave into a startup led second wave. Across these, the institutional context and interpretative package have been vital to the form this progress has taken. In closing, we also pointed to future interpretative and institutional potentials that could frame practices and outcomes in forthcoming third, or indeed fourth or fifth, waves of CM activity. We outlined these potential futures as well as possible concerns specifically in terms of corporate structures, environmental landscapes, and global meanings and infrastructures. We urge readers to be attentive to how cultural politics has reshaped CM, and how CM reshapes the cultural politics it inhabits. Such attention remains necessary when considering CM’s past, present, and future.

## Ethics Statement

The paper draws upon multiple independently conducted studies. Each study involved recorded interviews or focus groups with human subjects. NS 2010–2015 work was carried out in accordance with the recommendations of the Cardiff University School of Social Sciences Research Ethics Committee, with written informed consent from all subjects. NS 2018–2019 work was carried out in accordance with the recommendations of the Brunel University London College of Business, Arts and Social Sciences Research Ethics Committee. AS work was carried out in accordance with the recommendations of the King’s College London Ethics Committee, with informed consent from all subjects. CD work was carried out in accordance with the recommendations of the Wageningen School of Social Sciences.

## Footnotes

1 For technical reviews over time, see Edelman et al. (2005), Datar and Betti (2010), and Stephens et al. (2018).

2 The notion of an interpretative package was first developed in Gamson and Modigliani (1989).

3 Images of the feast are available here: http://www.tca.uwa.edu.au/disembodied/dis.html

4 See Dilworth and McGregor (2015) for discussion of early social science publications.

5 TEDMED talk available at https://www.youtube.com/watch?v=zDmkK8brSWk

6
https://www.crunchbase.com/organization/memphis-meats.

7 These comparisons were observed by Sexton during fieldwork visits in the San Francisco Bay Area in 2014-16.

8 See Sexton (2018) for more discussion.

9 Similar negotiations of sameness and difference have been examined by Sexton (2016) in the context of recent plant-based meat products.

10 See House (2016) for related discussion on edible insect products.

11 There are a diverse set of philosophical perspectives and empirical casestudies to support this understanding of meaning, including Wittgenstein (2009 [1953]) and Law and Singleton (2000); for food specific cases, see Roe (2006) and House (2018).

12 See, for example, the US Cattlemen’s Association’s (USCA) petition filed to FSIS, an agency of USDA (USCA, 2018).

13 For example, MEP Mara Bizzotto submitted a Parliamentary question to the European Commission in July 2018 on the potential health and economic risks ‘lab-produced synthetic meat’ poses to consumers and traditional meat producers (see http://www.europarl.europa.eu/doceo/document/E-8-2018-004200_EN.html).

14 The largest farm in the world is Mudanjiang City Mega Farm in China which manages around 100,000 dairy cows. The largest dairy farm in the US has 30,000 cows (Fair Oaks Farms), while Europe’s largest dairy farm reportedly under construction in 2017 had plans to house 20,000 cows (McShane, 2017).
